# Central Nervous System Cell-Derived Exosomes in Neurodegenerative Diseases

**DOI:** 10.1155/2021/9965564

**Published:** 2021-07-13

**Authors:** Yang Tian, Chen Fu, Yifan Wu, Yao Lu, Xuemei Liu, Yunling Zhang

**Affiliations:** ^1^Dongfang Hospital, Beijing University of Chinese Medicine, Beijing, China; ^2^Xiyuan Hospital, China Academy of Chinese Medical Sciences, Beijing, China

## Abstract

Exosomes are a type of extracellular vesicles secreted by almost all kinds of mammalian cells that shuttle “cargo” from one cell to another, indicative of its role in cell-to-cell transportation. Interestingly, exosomes are known to undergo alterations or serve as a pathway in multiple diseases, including neurodegenerative diseases. In the central nervous system (CNS), exosomes originating from neurons or glia cells contribute to or inhibit the progression of CNS-related diseases in special ways. In lieu of this, the current study investigated the effect of CNS cell-derived exosomes on different neurodegenerative diseases.

## 1. Introduction

Extracellular vesicles such as exosomes and microvesicles are released into the extracellular environment by various types of cells. The term “exosome” was first used for vesicles ranging between 40 nm and 1000 nm around 40 years ago. Since then, numerous advancements have isolated the secretion of a certain type of vesicle, located in reticulocytes, ranging from 40 nm to 100 nm, which was further associated with multivesicular endosome (MVE, also known as multivesicular body, MVB). On the other hand, microvesicles are now known to directly bud from the plasma membrane into the extracellular environment. Furthermore, recent studies on various cells, such as T cells, tumor cells, and CNS cells, have confirmed the release of a certain extracellular vesicle as a consequence of MVE fusion with the plasma membrane. The nomenclature “exosome” is now specific for 40 nm–100 nm extracellular vesicles which follow this particular generation pathway [1].

Current evidence further suggests that the formation of exosomes requires the involvement of multivesicular endosome (MVE). Certain stimulation has also been previously shown to cause plasma membrane “invagination,” such that the subsequently produced vesicle is coated by clathrin. Moreover, these clathrin-coated vesicles (CCV) are known to serve as a precursor of early endosomes. During development, the endosome membrane starts with invagination and forms an intraluminal vesicle (ILV). As a matter of fact, the extraendosome environment is the intracellular environment, and thus, the ILV contains diverse “cargo,” mRNAs, or small molecular proteins in its lumina or embedded in its plasma. The early endosome develops as MVE and reaches the point of divergence, such that some MVEs fuse with lysosome to digest the cargo, while other MVEs fuse with the membrane and release intraluminal vesicles into the extracellular environment and are now referred to as exosomes. The exosomes can now be uptaken by recipient cells and complete their mission of transferring from one secreting cell to another, and as a consequence, the exosomal inclusions such as proteins, mRNAs, miRNAs, and siRNAs, and membrane components such as membrane protein and lipids are transferred to the recipient cells in this process [2].

Neuronal loss, which may be caused by various of reasons, is a common occurrence in numerous neurodegenerative diseases such as Alzheimer's disease (AD), amyotrophic lateral sclerosis (ALS), Parkinson's disease (PD), Huntington's disease (HD), and multiple sclerosis (MS). Neuron loss is known to be triggered by different initial causes, undergo unique or shared pathogenic mechanisms, and finally lead to neuron degeneration. Meanwhile, other pathological developments, including inflammation, oxidative stress, excitatory toxicity, and abnormal autophagy, are always known to feature during these complex diseases. Unsurprisingly, various studies have highlighted the participation of exosomes in the aforementioned neurodegenerative diseases. While some exosomes have been documented to contain toxic cargos such as prion protein, amyloid precursor protein, superoxide dismutase 1 (SOD1), and alpha-synuclein [3, 4], some studies have also illustrated the protective functions of exosomes [5–7]. Meanwhile, other studies have even indicated alterations in exosomes in plasma and cerebrospinal fluid (CSF) as a potential biomarker in clinical practice. Exosomes were also found linked to multiple neurodegenerative diseases according to database-enabled analyses of comprehensively curated datasets [8]. In any case, it would be prudent to explore the role of the exosome in neurodegenerative diseases to better tackle these diseases.

**Figure 1:**
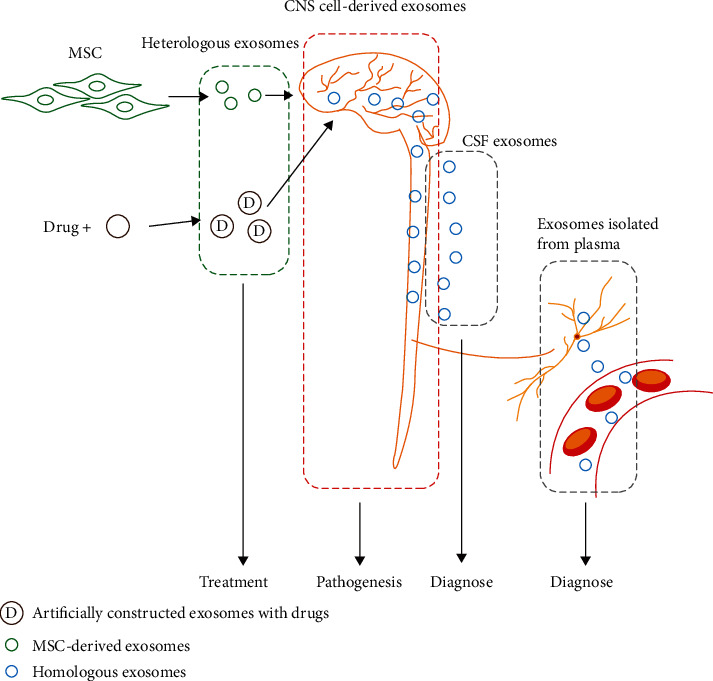
Description of different research area on exosomes in neurodegenerative diseases. The heterologous exosomes, including exosomes derived from MSC or served as drug delivery vehicle, were basically developed as treating purpose. The exosomes in CNS were basically found playing roles in disease pathogenesis. The exosomes isolated from plasma, or CSF was mainly developed for diagnosing purpose.

It is interesting to notice that exosomes have been found as “double-edge swords” in each of the diseases [4, 9–12]. These investigations, although similarly focused on exosomes, were actually carried out using different approaches, including the MSC or other stem cell-derived exosomes, exosomes serving as delivery vehicles after being conjugated with drugs, exosomes being isolated from CSF or plasma and studied as biomarkers, and the role of exosomes in the pathogenesis of neurodegenerative diseases (Figure 1). Meanwhile, the studies focusing on exosomes in CNS produced results lacking accuracy, and sometimes even contradicted each other, which is far from satisfying and convincing. Given the fact that the designs in a majority of these *in vivo* researches which directly isolate exosomes from CSF or tissue homogenate, while exosomes derived from different cells are known to exhibit different properties and functions, we think it is essential to highlight the type of secreting cells. Thus, the current study set out to categorize and present exosomes on the basis of neuronal or glial origin. Herein, identifying the origin of exosomes is highly possible to be the feature of studies in the next period.

## 2. Exosomes in CNS

As a pathway of cell-to-cell interaction, exosomes play an important role in interneuron or neuron-glia communication. Meanwhile, the secretion of neuronal exosomes is known to be altered by a range of factors. For instance, in cultured *γ*-aminobutyric acid (GABA) ergic neurons, incubation with GABA receptor was found to markedly augment exosomes secretion *via* the *α*-amino-3-hydroxy-5-methyl-4-isoxazolepropionic acid (AMPA)- and N-methyl-d-aspartate- (NMDA-) receptors-dependent pathway [13]. Similarly, increasing cytosolic calcium is also known to precipitate dramatically higher levels of exosomes' secretion. These exosomes can be transferred between spines of the same releasing neuron or to afferent neurons, wherein they exhibit involvement in controlling the physiology of neurons. It is likely that exosomes back-fuse with endosomes in recipient neurons, and the endosomes fuse with the plasma membrane and release the protein content from the releasing neuron on the surface, which serve as a way of regulating synaptic plasticity [14]. Furthermore, a research on human induced pluripotent stem cells (iPSCs) differentiated neurons has suggested that neuronal exosomes may carry signaling information for neurogenesis and circuit assembly [15].

Exosomes also possess the ability to participate in neuron-glia interaction and influence physiological trends. The study performed by Men et al. showed that *in vivo* neuronal exosomes transferred miR-124-3p to astroglia leading to higher expression of glutamate transporter 1 (GLT1), the predominant glutamate transporter [16]. Neuronal exosomes have further been shown to regulate complement levels in microglial cells and then facilitate synaptic pruning *in vitro* [17]. What is noteworthy is that neurons also interplay with oligodendrocyte *via* exosomes. Neurotransmitters are capable of stimulating oligodendroglial exosomes secretion, whereas neurons can internalize exosomes derived from oligodendrocytes and utilize the contents, which together highlights the involvement of exosomes in neuron-oligodendrocyte communication circuit [18].

Exosome-mediated delivery is gradually shown as a global and general way of normal intercellular communication in CNS cells. Exosomes also can be severely affected in disease condition and conferred their unique and essential roles in various stages of different neurodegenerative diseases.

## 3. CNS Cell-Derived Exosomes in AD

AD is the leading cause of dementia across the world and is characterized by the formation of amyloid plaques and neurofibrillary tangles (NFTs). The origin of amyloid plaques is attributed to the accumulation of misfolded amyloid-*β* protein (A*β*), including A*β*40 and A*β*42 [19]. A*β*42 exhibits a higher possibility of fibrillization and insolubility which lead to its higher abundance and carrying more blame for amyloid plaque formation. On the other hand, NFTs are caused by paired helical filaments composed of abnormally hyperphosphorylated Tau protein, such that both A*β* and hyperphosphorylated Tau are regarded as defining hallmarks of AD. It is interesting that the misfolded A*β* or Tau always starts from a focal region in the brain and then spreads to other areas, for instance, hyperphosphorylated Tau typically originates in the allocortex of the medial temporal lobe [20]. Both Tau and A*β* have been shown to spread across the synapses and self-propagate *via* the prion-like property [21].

An increasing number of studies have focused on the role of exosomes in the progression of AD. Exosomes are now known to contain and spread A*β*, as well as hyperphosphorylated Tau *in vivo* and *in vitro* [22–28]. Meanwhile, the levels of other proteins like synaptic protein AMPA4 and neurologin 1 (NLGN1) or lysosomal protein, and the expression of microRNAs were shown to be abnormal in plasma or CSF exosomes [29–32], which reiterates the involvement of exosomes in AD.

### 3.1. Neuronal Exosome in AD

The potential connection between A*β*42 and exosomes can be dated back to 2002, when Takahashi et al. illustrated the accumulation of A*β*42 in neuronal MVB, a key process in exosome biogenesis, in both rodent models and human brain tissues [33]. The accumulation in MVB located in both presynaptic and especially postsynaptic compartments is further associated with synaptic pathology. The study performed by Rajendran et al. confirmed this association by illustrating A*β*42 localization in isolated exosomes [34]. Meanwhile, exosomal proteins are also enriched in amyloid plaques, which is indicative of exosome contribution in A*β* propagation.

Additionally, Sinha et al. not only uncovered that the exosomes isolated from AD patients contain A*β* and are neurotoxic but also carried out a coculture experiment using SH-SY5Y which showed A*β* spread *via* exosomes and blocking formation, secretion, or uptake of exosomes could significantly reduce the toxicity *in vitro* [25]. Meanwhile, amyloid precursor protein (APP), namely, the protein-producing A*β*, is also known to be expressed in exosomes derived from human N2a cells. These APP-carrying exosomes can be endocytosed by recipient cells and deliver APP for further processes, which suggests that neuronal exosomes function as intercellular transport vehicles [35]. Moreover, another study demonstrated that A*β* is more likely to be associated with N2a-derived neuronal exosomes instead of glia-derived exosomes *in vitro* [36]. Collectively, either human sample or culture studies confirmed that A*β* is associated with neuronal exosome.

Furthermore, the Tau protein is known to be associated with neuronal exosomes in the pathogenesis of AD. Tau was previously found being released and trans-synaptically transmitted to cultured primary neurons or N2a cells *via* exosomes [37]. Meanwhile, exosomes isolated from neuronally differentiated, and human iPSCs were also found to express mutant Tau in another study. These exosomes could cause long-distance propagation of Tau pathology on mouse neurons *in vivo* [38]. However, Kanmert et al. argued that most of extracellular Tau is free-floating and unaggregated with only less than 1% Tau being carried by exosomes *in vitro*, which remains a point of controversy. Thus, it is remained to be further investigated and still hard to draw a conclusion on whether neuronal exosomes facilitate Tau propagation in real AD situations.

Nevertheless, various other proteins are known to be altered in exosomes compared to normal conditions. For instance, presynaptic protein, neuronal pentraxin 2 (NPTX2) and neurexin 2a (NRXN2a), and their respective postsynaptic functional partners, GluA4-containing glutamate (AMPA4) receptor and neuroligin 1 were all previously illustrated to be significantly decreased in neuronal exosomes derived from AD patients with cognitive loss [29, 30]. The synaptic protein loss in exosomes is suggestive of early excitatory circuit damage and, further, highlights the potential of exosomes as a biomarker in clinical practice.

### 3.2. Glial Exosomes in AD

Not only A*β* was found and established as the cargo in astrocyte-derived exosomes isolated from AD patients, the levels of *β*-site amyloid precursor protein-cleaving enzyme 1 (BACE-1), *γ*-secretase, soluble A*β*42, soluble amyloid precursor protein (sAPP)*β*, sAPP*α*, glial-derived neurotrophic factor (GDNF), P-T181-Tau, and P-S396-Tau were all found to be higher than the normal situation, even higher than the levels in neuronal exosomes [39]. Astroglial exosomes also possess the ability to facilitate the aggregation of A*β* and interfere with glial uptake *in vitro* [40]. Furthermore, another study documented significantly higher levels of complements including C1q, C4b, C3d, factor B, factor D, Bb, C3b, and C5b-C9 terminal complement complex in astrocyte-derived exosomes of AD patients compared to the control group after normalization with exosomal marker CD81. Similarly, normalized Interleukin-6 (IL-6), tumor necrosis factor-*α* (TNF-*α*), and IL-1*β* were all found to be dramatically higher in astrocyte exosomes for AD patients relative to normal control, which indicates the participation of astroglial exosomes in inflammation in AD [41]. The aforementioned evidence suggests that either higher complements or the association with inflammatory factors worsen neuron damage.

Moreover, Asai et al. [42] illustrated the ability of microglia to phagocytose Tau and secret it *via* exosomes *in vitro*. Similarly, depleting microglia is also known to halt Tau propagation *in vivo*. By combining them together, it is reasonable to argue that Tau migration in AD disease partially undergoes a microglial exosome-dependent mechanism. It is also noteworthy that the potential association between microglia and A*β* has previously been highlighted in another research [27]. The authors figured out that exosomes targeted microglia preferentially *in vivo*, such that activated microglia could engulf the exosomes clustering around the A*β* plaques in the meantime. Altogether, the evidence suggests that exosomes play a role in Tau deposition *via* microglia.

## 4. CNS Cell-Derived Exosomes in PD

PD, the second most common neurodegenerative disorder, clinically featured by the classical Parkinsonism movement disorder, has long been characterized by the formation of Lewy bodies and loss of dopaminergic motor neurons especially in the substantia nigra from a pathological aspect. Although the pathology of PD now is regarded as heterogenous other than just Lewy bodies, the presence of Lewy bodies is still considered as a hallmark of PD [43, 44]. The occurrence of Lewy bodies is attributed to the aggregation of various proteins with *α*-synuclein (*α*-syn) being the major constituent, and not only responsible for PD but also featured in other neurological disorders such as AD and Lewy body dementia [45]. The progression of PD commonly, not necessarily, starts from the brain stem, then leading to midbrain lesion, and finally precipitates severe involvement of the brain including neocortical areas. The progression is correlated with *α*-syn migration, which is also considered as a prion-like cell-to-cell transmission [46]. Interestingly, transneuronal propagation of *α*-syn was previously documented in peripheral areas, such as the olfactory bulb or gut, to brain regions, including substantia nigra *in vivo* [47, 48]. Recent studies have further highlighted the correlation between *α*-syn and exosomes both *in vivo* and *in vitro* [49, 50]. Meanwhile, exosomes isolated from PD patients were previously illustrated to contain a pathologic species of *α*-syn which was capable of conferring disease pathology to recipient cells [51]. On the other hand, exosomes are also known to confer protective effects in PD [52]. Thus, understanding CNS cell-derived exosomes is prudent for further research in PD progression.

### 4.1. Neuronal Exosomes in PD

In cultured SH-SY5Y cells, exosome-mediated *α*-syn were found to be transferred from releasing cells which overexpressed *α*-syn to normal recipient SH-SY5Y, promoting the process of lysosome dysfunction [49, 53]. Moreover, Mn^2+^ exposure is known to augment *α*-syn secretion in exosomes and, further, lead to more severe dopaminergic neurotoxicity both *in vivo* and *in vitro* [54]. However, the association is not always regarded as a harmful mechanism. It was formerly shown that macroautophagy could degrade intracellular *α*-syn and reduce the toxicity. After blocking this protective mechanism, increased secretion of *α*-syn in exosomes arose as a compensatory mechanism for aggregated protein disposal and is vital for cell viability in the absence of autophagy. Meanwhile, depleting exosomes by GW4896, a neutral sphingomyelinase (nSMase) inhibitor that blocks release of exosomes, was previously shown to cause heavier cell death in this model [55].

Exosomes are also highly implicated in the interfere of neurons exerted on glia cells. The overexpression and accumulation in SH-SY5Y of *α*-syn could impair microglial autophagy function *via* exosomes containing higher levels of miR-19a-3p [56]. Meanwhile, the exosome-dependent *α*-syn transfer from neurons, exposed to methamphetamine can increase *α*-syn levels, to astrocyte was able to cause inflammatory response *in vitro* [57].

Although it seems rational to correlate *α*-syn with neuronal exosomes in light of experiments carried out on cultured neuroblastoma cell line, the fact that exosomes isolated from human iPSCs-derived dopamine neurons did not exhibit association with extracellular *α*-syn [58] challenges this view. There is a dearth of *in vivo* evidence to argue the exact relationship of neuronal exosomes and disease progression, which highlights the need to elucidate the role of neuronal exosomes in the disease pathogenesis of PD in future studies.

### 4.2. Glial Exosomes in PD

Although *α*-syn predominantly exists in neurons, glia cells have been found to be tightly involved in *α*-syn aggregation pathology [59–61].

Microglial exosomes are capable of facilitating *α*-syn and inducing protein aggregation *in vitro*. Meanwhile, exogenous exosomes are known to dysregulate autophagy of the BV2 microglia cell line, resulting in increased accumulation of intracellular *α*-syn and accelerated secretion of *α*-syn into extracellular space [62]. It is also noteworthy that aggregation increased when microglial exosomes were combined with proinflammatory cytokines. Similarly, the toxicity of exosomes isolated from *α*-syn preformed fibrils-treated microglia recapitulated the result above *in vivo* as well. Colocalization of CD11b^+^ exosomes with *α*-syn oligomer has also been previously documented in CSF of PD patients [59].

Furthermore, astrocyte-derived exosomes have been indicated to be potentially beneficial in PD, as they contain miR-200a-3p and prevent SH-SY5Y cell death induced by MPP^+^, a common neurotoxin to mimic PD models [63]. As for oligodendrocyte-derived exosomes, reduced secretion is regarded as an important mechanism in the aggregation of *α*-syn in multiple system atrophy, an *α*-syn involved disease like PD. It is likely that exosomes secreted from oligodendrocyte could undergo the same mechanism in PD, but there is no direct evidence to support this hypothesis at the moment [64].

## 5. CNS Cell-Derived Exosomes in ALS

Amyotrophic lateral sclerosis (ALS) is a disease characterized by the loss of both upper and lower motor neurons, yet the cause of ALS remains unknown. Nevertheless, the C9orf72 mutation has been found as the most common mutation with around 20~40% occurrence rate in familial ALS, followed by superoxide dismutase 1 (SOD1) mutation with 5~20% rate, TAR DNA-binding protein (TDP43) mutation with 5~10% rate, and gene encoding fused in sarcoma (FUS) mutations with 5% amongst various others [65–67]. Meanwhile, another study investigated the C9orf72 mutation model and found that motor neuron degeneration may be a subsequent of loss of C9orf72 protein function, gain of toxicity of C9orf72 RNA, or toxic dipeptide produced by mutant hexanucleotide repeat expansion [68]. As for SOD1 mutation, it was found that gain of toxicity may lead to inflammation, excitatory toxicity, and so on, instead of the influence on the SOD1 enzyme. Misfolded SOD1 can also undergo a prion-like progress, almost like the Tau protein, which allows the misfolded SOD1 to transfer to the recipient cell as a seed, and further trigger aggregation and more misfolding. Meanwhile, mutant TDP43 protein can also increase the propensity to aggregate, enhance cytoplasmic mislocalization, alter protein stability, resistance to proteases, or modified binding interactions with other proteins [69].

ALS pathologic proteins were first associated with exosomes in 2007, wherein SOD1 was found to be associated with exosomes derived from neuron-like NSC-34 cells which confirmed SOD1 secretion *via* exosomes [70]. Since then, multiple major pathologic proteins, including SOD1, toxic dipeptide repeat proteins (DRPs) transferred from mutate C9orf72, TDP43, and FUS, have been determined to be associated with exosomes in cell lines, in rodent models, and in human tissues [71–75]. Moreover, the exosomal gene or proteomic expression levels are also known to be altered in ALS patients [75–77]. Even though the very original pathogen in ALS remains to be further investigated, it seems plausible to suggest that exosomes are surely involved in ALS progression based on existing evidence.

### 5.1. Neuronal Exosomes in ALS

In 2007, Gomes et al. employed mouse NSC-34 cells overexpressing human wild-type SOD1 or mutant SOD1 and uncovered SOD1 colocalized with the exosomal marker CD9 in pellets after 100,000 x g centrifugation, which revealed SOD1 could be secreted *via* motor neuron-like cell-derived exosomes [70]. A subsequent research has demonstrated that exosomes derived from SOD1-transfected NSC34 can be uptaken and trigger aggregation in recipient cells, which indicated that misfolded SOD1 can be intercellularly transferred *via* an exosome-dependent mechanism [78]. Using the same *in vitro* models, Pinto et al. found that miR-124 was also enriched in these exosomes derived from NSC-34 transfected with hSOD1-G93A. Meanwhile, NSC-34 was previously shown to induce cocultured N9 microglial cells to be more senescent-like positive *via* these miR-124 enriched exosomes [79]. However, the aforementioned experiment seems to be controversial to the work showing that miR-124-3p was actually decreased in spinal neurons *in vivo* [80], which can precipitate lower GLT1 expressions in ALS [81]. However, the work of Yelick et al. [80] demonstrated an increased association of miR-124-3p with spinal cord motor neuron-derived exosomes, which is a possible explanation to the contradiction. Furthermore, *in vivo* experiments have also illustrated that SOD1 was carried by astrocytes and neuron-derived exosomes in CNS [71].

Neuronal exosomes are also known to carry TDP43 and DRPs. Interestingly, the intercellular transmission of pathological DRPs can undergo exosome-dependent pathway, synaptic-dependent pathway, or extracellular but exosome-independent pathway. Moreover, DRPs isolated from DRP transfected NSC34 cells were previously illustrated to be colocalized with exosomes and could be transferred into cultured neurons or cortical neurons [82]. On the other hand, TDP43 was revealed to be transmitted in MVE in HEK293 cells using *in vitro* studies [83]. It was also found that phosphorylated TDP43 can propagate and aggregate between cultured SH-SY5Y cells which may partially depend on exosomes [74]. However, another research evidenced that secreting exosomes could be a key pathway for toxic TDP43 clearance. However, this particular research only detected TDP43 from neuronal exosomes instead of astro- or microglial exosomes, which might explain the opposing views. Furthermore, inhibiting exosomes by GW4896 is known to provoke TDP4 aggregation in N2a cells, which suggests that neuronal exosomes overall confer a beneficial role *in vivo* [84].

### 5.2. Glial Exosomes in ALS

We have already established that exosomes isolated from the mouse brain or spinal cord contain misfolded SOD1. Moreover, flow cytometric experimentation has shown that these exosomes were primarily derived from astrocytes and neurons instead of microglia or oligodendrocytes, which indicates that the majority of the CNS cell-derived exosomes *in vivo* were secreted by astroglia and neuron and facilitated the spread of toxic misfolded SOD1 [71]. While misfolded mutant SOD1 is recognized as a cargo, the miRNA levels are known to be aberrantly different from normal conditions in mice [85, 86], and IL-6 level was higher in patients [87], in astroglial exosomes.

Meanwhile, in terms of microglia, SOD1 secretion is known to be partially dependent on exosomes [88]. Activated N9 microglial cells were further unraveled to possess the ability to secrete SOD1 *via* exosomes *in vitro*. Interestingly, exosomes derived from wild-type SOD1 treated N9 do not overexpress miR-146a like the condition in cells; instead, the exosomes from mutant SOD1-treated N9 exhibited higher miR-155/miR-146a levels which recapitulated the cells [89]. However, the experiments are controversial to the evidence that microglia barely secrete exosomes *in vivo* [71]. Given the fact that these experiments on microglia were carried out *in vitro*, it is likely that the distinction is occurred due to the difference between internal and external experiments.

## 6. CNS Cell-Derived Exosomes in HD and MS

Huntington's disease (HD) is a progressive neurodegenerative disease caused by expanded CAG mutation in the Huntington gene (HTT), which leads to an expanded polyglutamine stretch in the huntingtin protein (Htt) [8, 90]. Multiple sclerosis (MS) is a disease characterized by perivenular inflammatory lesions, leading to demyelinating plaques, although the primary cause of immune response still remains elusive [91, 92]. Unfortunately, only a handful of studies have focused on HD or MS with exosomes. One such study elucidated that exosomes can transfer miR-124 [93] or mediate the delivery of hydrophobically modified siRNA in HD *in vitro* [94]. Furthermore, alterations in miRNA profiling have been documented in exosomes harvested from MS patient blood samples, which suggests that serum exosomes can be potentially developed as a tool to monitor MS [95–97]. However, there is no direct evidence linking HD or MS with CNS cell-derived exosomes or showing how exosomes contributed in the propagation of these diseases.

## 7. Conclusion and Perspectives Section

Ever since the discovery of exosomes, a plethora of studies has investigated the role of exosomes in neurodegenerative diseases. It is well-established that exosomes can serve as biomarkers or therapeutic drug-delivery systems in clinical use for numerous neurodegenerative diseases [12, 98–102]. The abundant cargo contained in exosomes originates from cells and surely is influenced by disease situation and, collectively, provides enough targets for testing, which reiterates the potential of exosomes as clinical biomarkers. Inherently, exosomes are nanosized vesicles naturally secreted by cells, uptaken by cells, capable of crossing the blood-brain barrier, and further exhibit immune-privileged status that can efficiently decrease drug clearance, which together makes exosomes ideal for delivering drugs for CNS diseases [100, 103]. Furthermore, a number of studies have uncovered the protective effect and possible therapeutic application of exosomes in AD or PD [5–7].

**Figure 2:**
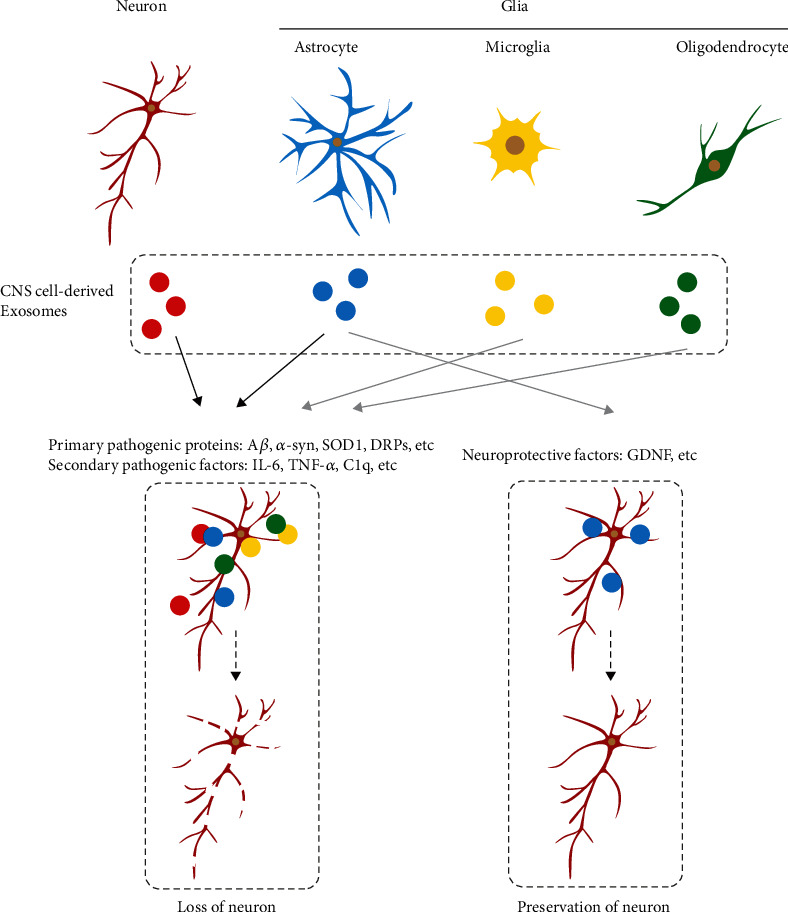
Description of basic effect of cell type-specific exosomes on neurodegeneration. A certain color of exosomes represents that they are derived from the cell type with the corresponding color. Grey arrow means that no enough evidence or evidences contradicting to each other to draw the conclusion. Neuron or glia-derived exosomes could facilitate neuron degeneration. Astroglial exosomes may have neuroprotective property. The arrow of “microglial exosomes” to “loss of neuron” is marked grey since some evidence showed that microglia may not secret exosomes. The arrow of “oligodendrocyte-derived exosomes” to “loss of neuron” in marked grey since no enough researches focused on it. The arrow of “astrocyte-derived exosomes” to “preservation of neuron” is marked grey since no enough research supporting it.

The current study set out to elucidate the role of different cell-derived exosomes in neurodegeneration. Roughly speaking, most CNS cell-derived exosomes serve as an approach for the spread of pathological factors in diseases and, thus, should be recognized as neurotoxic. It is noteworthy that exosomes carry matters that originated from releasing cells, which definitely turn them into vehicles of A*β*, *α*-syn, SOD1, DRPs, and other inflammatory factors or complements triggered by primary pathological proteins, and consequently lead to neurodegeneration. Despite the toxic nature of cargos in exosomes, astrocyte-derived exosomes have been previously highlighted to exert neuroprotective effects in certain conditions [39]. It is common knowledge that astrocytes are the supporting cells which provide structural and metabolic support to neurons [104]. Meanwhile, it is also recognized that exosomes facilitate the transport of miRNA-124 from neurons to astroglia and, further, lead to higher GLT1 expressions [81]. It is thus reasonable to speculate that the global effect of astroglial exosomes may be neuroprotective in nature, which is in accordance with another study that highlighted that astrocyte-derived exosomes contain GDNF [39]. On the other hand, microglia are well-recognized as the resident macrophage of the CNS. The phenotype of microglia can be generally described as neuroprotective, which is induced by toll-like receptors or interferon *γ*, or neuroinflammation induced by IL-4 or IL-13. Moreover, the hard done work of our peers has shown that microglia do contribute to disease progression in chronic neurodegenerative diseases [105]. Furthermore, previous findings reveal that microglia-derived exosomes contain complements, inflammation factors, and proinflammation factors, which highlights their neurotoxic function in neurodegenerative diseases, which is consistent with the function of microglia. The real global challenge, elucidating the role of cell type-specific exosomes in neurodegenerative diseases still requires more specific and well-organized experiments (Figure 2).

With growing body of works on exosomes, we certainly understand them more, but there are still blanks to fill and questions to answer. First of all, it could be very important to precisely determine the function of exosomes originated from certain cell type, which is the prime focus of the current study. A vast number of *in vivo* studies have been following a set pattern of design: isolating exosomes by ultracentrifuge from tissue homogenate and testing the function of these exosomes irrespective of the variations in releasing cells. This approach can surely answer some questions, but it fails to provide precision. Researchers in the past have had to employ this approach since there is no proper way to mark cell type-specific exosomes, but a new experimental design and way of application such as those in Men or Silverman's work appeared to be groundbreaking [16, 71]. The differences in origins of exosomes should be clearer, and then, it would make more sense to discuss which exosomes are toxic, which exosomes are beneficial, and which exosomes really portray the “double-edge sword” role.

In the meantime, when talking about the fact that exosomes can facilitate the diffusion of toxic factors in neurodegenerative diseases, it is essential but also challenging that the ratio of certain pathologic protein associated with exosomes should be determined, quantified, and compared to the ratio of exosome-independent transporting protein. For instance, a recent search using healthy N2a cultures illustrated that less than 1% of extracellular Tau exists in exosomes, while the majority of Tau remains free-floating [106]. Future studies should investigate if this ratio is altered in diseased conditions. If the ratio remains the same, it would be less suitable to argue the importance of exosomes in the migration of Tau, but if the ratio is changed, that would be promising.

What is more, we came across some controversial conclusions during the course of the current study. Taking microglial exosomes as an example, multiple studies have confirmed the basic fact that microglia secrete exosomes; however, Silverman et al. showed that CNS-derived extracellular vesicles were primarily derived from neurons or astrocytes, instead of microglia *in vivo* [71]. The variations in these results may be attributed to design flaws in Silverman's work, but most likely would construe from the difference between *in vivo* and *in vitro* findings. It is less appropriate to argue the property of microglia based on cultured cell lines or primary microglia comparing to *in vivo* study. Thus, more *in vivo* studies on cell type-specific exosomes should be carried out to mimic disease situations in humans as much as possible and further investigate their real role in that condition. Moreover, new techniques have enabled researchers to identify cell type-specific exosomes isolated from patients [107], which also helped us to understand the underlying functions of exosomes in these diseases.

While plenty of works have explored the association of exosomes with neurodegenerative diseases, the role of CNS cell-derived cell type-specific exosomes requires further investigation. Further *in vivo* and *in vitro* findings and data of exosomes from patients shall give us a more comprehensive understanding of the same.

## Abbreviations

*α*-syn: *α*-Synuclein
AD: Alzheimer's disease
ALS: Amyotrophic lateral sclerosis
AMPA4: GluA4-containing glutamate
APP: Amyloid precursor protein
A*β*: Amyloid-*β* protein
BACE-1: *β*-Site amyloid precursor protein-cleaving enzyme 1
CCV: Clathrin-coated vesicles
CNS: Central nervous system
CSF: Cerebrospinal fluid
DRPs: Dipeptide repeat proteins
GDNF: Glial-derived neurotrophic factor
GLT1: Glutamate transporter 1
HD: Huntington's disease
Htt: Huntingtin protein
HTT: Huntington gene
IL-6: Interleukin-6
ILV: Intraluminal vesicle
iPSC: Induced pluripotent stem cell
MS: Multiple sclerosis
MSC: Mesenchymal stem cell
MVB: Multivesicular body
MVE: Multivesicular endosome
NFTs: Neurofibrillary tangles
NPTX2: Neuronal pentraxin 2
NRXN2a: Neuronal pentraxin 2a
nSMase: Neutral sphingomyelinase
PD: Parkinson's disease
SOD1: Superoxide dismutase 1
TNF-*α*: Tumor necrosis factor-*α*.
